# Fungal Malignant Otitis Externa with Facial Nerve Palsy: Tissue Biopsy Aids Diagnosis

**DOI:** 10.1155/2014/192318

**Published:** 2014-02-05

**Authors:** Jenny Walton, Chris Coulson

**Affiliations:** Department of Otorhinolaryngology, Queen Elizabeth Hospital Birmingham, Mindelsohn Way, Edgbaston, Birmingham B15 2WB, UK

## Abstract

Fungal malignant otitis externa (FMOE) is a serious and potentially life-threatening condition that is challenging to manage. Diagnosis is often delayed due to the low sensitivity of aural swabs and many antifungal drugs have significant side effects. We present a case of FMOE, where formal tissue sampling revealed the diagnosis and the patient was successfully treated with voriconazole, in addition to an up to date review of the current literature. We would recommend tissue biopsy of the external auditory canal in all patients with suspected FMOE in addition to routine microbiology swabs.

## 1. Introduction

Malignant otitis externa (MOE) remains a relatively uncommon condition but can lead to serious morbidity or mortality as diagnosis is often delayed leading to initially ineffective treatment [1]. The infection begins in the external auditory canal, typically presenting with severe otalgia and purulent otorrhoea; it can rapidly spread via the ear canal soft tissue to the temporal bone resulting in osteomyelitis and subsequent cranial nerve palsies and intracranial infection. The prevalence of bacterial MOE is not accurately documented [2] and it is unknown whether fungal MOE is a repercussion of unsuccessfully treated bacterial otitis externa or if it represents a *de novo* presentation of fungal disease.

The most commonly reported pathogen in malignant otitis externa is *Pseudomonas aeruginosa* [2]. *Aspergillus *species and *Candida albicans* have been implicated in fungal MOE. There are 32 previously reported cases of fungal MOE, usually occurring in patients with some form of immunosuppression—typically diabetes, acquired immunodeficiency, or malignancy. We present a case of fungal malignant otitis externa complicated by a facial nerve palsy that proved very difficult to achieve a formal diagnosis.

## 2. Case

An 83-year-old man with type 2 diabetes presented with right sided otalgia and otorrhoea; examination revealed granulation tissue inferiorly in his ear canal at the Osseochondral junction. A clinical diagnosis of malignant otitis externa was made. *Pseudomonas aeruginosa* was grown on microbiological culture of the discharge, sensitive to ciprofloxacin. His condition was successfully treated over a period of three months with oral and topical ciprofloxacin and he was discharged from clinic pain-free with an otoscopically normal canal.

One year later, he presented with left-sided grade III facial nerve palsy and a painful left ear (note this is the contralateral side to the original presentation). The left ear was initially otoscopically normal. CT and MR imaging demonstrated a left skull base osteomyelitis with involvement of the temporal bone and extension to the clivus with an associated small epidural abscess. Over the next few months, the VII nerve palsy progressed to complete paralysis (grade VI) and was unresponsive to treatment with ciprofloxacin and meropenem. He developed inflammation of his left external canal with an associated otorrhoea. Numerous aural swabs revealed no definitive microbiological diagnosis. Tissue biopsies were taken directly from the canal and, in contrast to the previous microbiology, *Aspergillus flavus* was isolated. Treatment was commenced with intravenous voriconazole for 2 weeks, followed by subsequent conversion to oral voriconazole. Within a month of commencing antifungal therapy, he became pain-free and his facial palsy resolved completely. Oral voriconazole therapy was continued for 10 months after resolution of symptoms. The initial plan had been to treat him for 12 months, although the patient developed worsening liver function; hence, the therapy was discontinued.

## 3. Discussion

Cranial nerve palsies are frequently seen in fungal MOE [4]; however, many reported cases have not demonstrated a significant improvement following treatment, possibly due to the delayed isolation of the pathogen and initiation of antifungal treatment. Halsey et al. [4] reported the presence of VII nerve palsy in 75% of patients with *Aspergillus* infection, compared with only 34% in MOE due to *Pseudomonas*.

*Aspergillus fumigatus* was thought to be the most common fungal pathogen in MOE [6], with *Aspergillus flavus* assumed to be a less frequent cause of the condition. However, review of published cases reveals *A. flavus* to be a roughly as prevalent as *A. flavus* in MOE. *A. flavus* has been observed to be 100 times more virulent than *A. fumigatus* and has an optimum temperature for growth of 37°C, which may explain its particular pathogenicity in humans [29].

In MOE, the infection spreads through the fissures of Santorini, small perforations in the cartilaginous floor of the external auditory canal, and then medially until it reaches the skull base [1]. Here, it causes bony destruction and further continues its medial progression triggering cranial nerve palsies with the VII nerve being most commonly affected due to the proximity of the stylomastoid foramen to the ear canal. The presence of a facial nerve palsy has been suggested to represent a poor prognosis in patients with MOE along with coexisting immunosuppression [30].  Table 1 summarizes the cases of fungal MOE (FMOE) present in the literature.

## 4. Summary of the Literature

The mean age of patients in the literature was 52 years with a male : female ratio of 7 : 3. *Aspergillus* species were the most commonly implicated pathogens. The most frequently occurring comorbidity was diabetes mellitus but haematological malignancy and acquired immunodeficiency were also recognized. 44% (14/32) of patients were reported to have an associated cranial nerve palsy and 29% (4/14) of these did not resolve following treatment. There was limited information on total duration of treatment for most of the reported cases. Amphotericin B and itraconazole were favoured for treatment of FMOE in the earlier case reports, whereas voriconazole has played a role in the treatment of the majority of reported cases since 2008. The mortality rate of FMOE was 15% (5/32).

Voriconazole is currently recommended as first line treatment in cases of invasive aspergillosis [31] and its use is increasing since its launch in 2002. The intravenous form is recommended for use in systemically unwell patients, with the oral form being reserved for those who are stable or have improved following initial intravenous treatment. Voriconazole is widely distributed throughout tissues and, in its oral form, is not usually associated with worsening of renal function. This is particularly important as it is often patients with comorbidities affecting renal function such as diabetes, who develop aspergillosis FMOE and therefore require treatment with voriconazole.

The most commonly reported side effects of voriconazole include visual disturbance, particularly to colour vision, abnormal liver function tests, deranged renal function, and electrolyte abnormalities [32]. As noted in the literature, amphotericin B was most frequently used, although it has a significantly poorer side effect profile, including derangement of renal function [5]. Itraconazole and caspofungin have also been used in cases reported in the literature.

Antibiotic therapy for the majority of cases of MOE is guided by the results of aural swabs sent for microbiological culture. Diagnosis of FMOE in our case and other cases in the literature required formal tissue sampling from the external auditory canal in order to identify the pathogen [4, 20, 33].

The patient in our case presented one year following treatment of bacterial MOE with symptoms in the contralateral ear. Although we found no evidence that the causative organism was the same on both sides, we note one particular case in the literature where the disease spread from one side to the other via the clivus [31].

## 5. Conclusion

We would recommend biopsy of tissue from the external auditory canal in any patient with a presumed diagnosis of MOE in addition to aural swabs. The importance of this is to obtain early confirmation of a fungal pathogen thereby allowing timely treatment to be commenced.

## Figures and Tables

**Table 1:** Summary of published cases of FMOE. M: male, F: female, DM: diabetes mellitus, N: no, Y: yes, NIDDM: noninsulin-dependent diabetes mellitus, AML: acute myeloid leukaemia, tx: transplant, CRF: chronic renal failure, HTN: hypertension, AIDS: acquired immunodeficiency syndrome, and ALL: acute lymphocytic leukaemia. Numbers in round brackets represent separate patients reported in the same paper.

Main author	Year of paper	Age (years)	M/F	Relevant comorbidities	Pathogen	Nerve palsy	Treatment	Surgical debridement	Symptom resolution
Tarazi et al. (1) [3]	2012	77	M	Type 2 DM (poorly controlled)	*Aspergillus *	No	Amphotericin then voriconazole	N	Y
Tarazi et al. (2) [3]	2012	85	F	Type 2 DM (poorly controlled)	*Aspergillus *	VII	Amphotericin then voriconazole	N	Y
Tarazi et al. (3) [3]	2012	70	F	Type 2 DM (poorly controlled)	*Aspergillus *	No	Voriconazole	N	Y
Halsey et al. [4]	2011	62	M	AML	*Aspergillus wentii *	VII	Amphotericin B then itraconazole	Y	No—CN palsies persisted
Parize et al. (1) [5]	2009	48	M	Polychondritis	*Aspergillus niger *	No	Voriconazole	N	Yes
Parize et al. (2) [5]	2009	40	F	IDDM, renal tx, CRF	*Aspergillus niger *	No	Voriconazole	Y	N—hearing loss
Ling and Sader [6]	2008	77	M	NIDDM, HTN, gout	*Candida, Aspergillus flavus *	VII	Voriconazole, caspofungin	Y	Yes
Narozny et al. [7]	2006	65	M	NIDDM	*Aspergillus *	VII, IX, X, XII	Amphotericin B, itraconazole	Y	N—died
Tzuku et al. [8]	2006	17	F	IDDM	*Scedosporium apiospermum *	No	Amphotericin B	Y	No—died on day 7
Bellini et al. [9]	2003	73	M	NIDDM	*Aspergillus niger *	Unspecified	Amphotericin B then itraconazole	N	Yes
Shelton et al. [10]	2002	58	M	Nil	*Aspergillus niger *	Unspecified	Itraconazole	N	Yes
Finer et al. [11]	2002	7	M	Neuroblastoma	*Aspergillus flavus *	Unspecified	Amphotericin B then itraconazole	N	Yes
Chai et al. [12]	2000	53	M	NIDDM	*Malassezia sympodialis *	VII, IX, X, XII	Amphotericin B then fluconazole	N	No—CN palsies persisted
Chen et al. (1) [13]	1999	41	M	AIDS	*Aspergillus fumigatus *	VII	Amphotericin B then itraconazole	Y	Yes
Chen et al. (2) [13]	1999	36	M	AIDS	Unknown	VII	Amphotericin B	Y	No—CN palsies persisted
Slack et al. [14]	1999	14	F	ALL	*A. flavus, niger, fumigatus *	Unspecified	Amphotericin B then itraconazole	Y	Yes
Muñoz and Martínez-Chamorro [15]	1998	27	F	AIDS	*Aspergillus fumigatus *	Unspecified	Itraconazole then amphotericin B	N	N—died
Ress et al. [16]	1997	41	M	AIDS	*Aspergillus fumigatus *	Unspecified	Timentin-gentamicin	N	N—died
Kountakis et al. [17]	1997	65	M	NIDDM	*Aspergillus flavus *	VII	Amphotericin B	Y	No—CN palsies persisted
Yates [18]	1997	18	M	AIDS	*Aspergillus fumigatus *	Unspecified	Itraconazole	Y	Yes
Harley et al. [19]	1995	20	M	ALL	*Aspergillus flavus *	VII	Amphotericin B	Y	N—died
Gordon and Giddings (1) [20]	1994	67	F	NIDDM, HTN	*Aspergillus flavus *	No	Amphotericin B then itraconazole	Y	No
Gordon and Giddings (2) [20]	1994	82	M	Nil	*Aspergillus flavus *	No	Ceftazidime, ciprofloxacin, tobramycin, itraconazole	Y	No
Hanna et al. [21]	1993	61	M	NIDDM	*Aspergillus flavus, Candida *	VII	Amphotericin B	Y	Yes
Reiss et al. (1) [22]	1991	42	M	AIDS	*Aspergillus fumigatus *	Unspecified	Amphotericin B then itraconazole	Y	Yes
Reiss et al. (2) [22]	1991	30	M	AIDS	*Aspergillus fumigatus *	Unspecified	Amphotericin B then itraconazole	Y	Yes
Phillips et al. [23]	1990	64	F	NIDDM, AML	*Aspergillus *	VII	Amphotericin B then itraconazole	N	Yes
Menachof and Jackler [24]	1990	46	F	AML	*Aspergillus flavus *	VII	Amphotericin B	Y	Y
Denning et al. [25]	1989	38	M	Nil	*Aspergillus fumigatus *	No	Itraconazole	Y	Yes
Cunningham et al. [26]	1988	85	M	Nil	*Aspergillus fumigatus *	VII	Amphotericin B, rifampicin	Y	Yes
Bickley et al. [27]	1988	80	M	Myelodysplasia	*Aspergillus *	No	Amphotericin B	Y	Yes
Petrak et al. [28]	1985	68	M	AML	*Aspergillus fumigatus *	VII	Amphotericin B	Y	No—CN palsies persisted
